# The Problem of Repopulation

**Published:** 1917-01-13

**Authors:** 


### The Hospital

The Workers' Newspaper of Administrative Medicine and Institutional Life, Administration, National Insurance and Health.

No. 1597, Vol. LXI. SATURDAY, JANUARY 13, 1917. PRICE ONE PENNY

### THE PROBLEM OF REPOPULATION.

For the next quarter of a century almost every European country, not to speak of Canada and Australia, will be faced with a great deficit in, at any rate, its population of healthy male adults. What makes this fact the more serious is that, even without and before the slaughter of the war, most of the present belligerent States, except, probably, Russia, experienced already the same difficulty, although, of course, in much lesser degree than now. In the case, however, of France it was bad enough then. But after two years of high explosives, what with the loss by death and by withdrawal from the country of men of reproductive age; what with the crippling of many of those who return, unfitting them for the burden of keeping a family; and what with some extra prevalence of venereal disease (the pest of all armies), with its sterilising or deteriorating effect, the inevitable further fall in the European birth-rate must as inevitably induce practical measures of "eugenics" in the widest connotation of that term. Spite of the unsurpassable distraction of the greatest war in history, such measures have already begun to be discussed. Here, in conformity with the spirit of action everywhere invoked, let us notice such practical recommendations as have already been made.

On those of the body which deliberated the subject in this country some time ago all we need say is that the contradiction of their conclusions made by Dr. Havelock Ellis does not seem altogether justified. Dr. Ellis' rare breadth and height of outlook, both natural and acquired by a lifetime's study of the highest quality, have even perhaps led him astray. It may be that the decline in the human birth-rate is a natural phenomenon, conformable with the law that the more complicated an organism is in development and structure and conditions of life, the fewer its progeny tends to be. But that decline began rather suddenly; and in any case *laissez-faire* deductions based on this biological argument must be vitiated by an artificial factor of immense immediate potency—namely, such a wholesale killing of desirable fathers as has never before been known. Something to countervail must be put in action, or the highest existing civilisation may fall before Asiatics.

To begin, then, with France, whose strait is the worst of all. M. Bénazet has put before the French Chamber the following proposed enactments: The State to give every mother 500 frs. apiece for her two first living children, 1,000 frs. for the third, 2,000 frs. for the fourth, and for each succeeding child 1,000 frs. These moneys to be the exclusive property of the mother, and to be paid her (a year after the respective confinement), indifferently whether she be married or single. The father would be rewarded with a premium of 2,000 frs., but only provided that he could show four living children (the size of family that the author of these proposals considers necessary to the maintenance of the race), whom he had supported uninterruptedly from birth onwards. The funds for all these payments are to be got by taxing childless persons, and families with only one child.

In Germany a society (*Deutsche Gesellschaft für Bevölkerungspolitik*) has been formed for study of the subject, and in this quarter and others many of its details considered. The society has proposed the suppression of all advertisements of anticonceptional (except those involving protection from venereal infection) and abortive appliances. The latest Rector of Berlin University appears to be a gynæcologist, and in his inaugural address he deprecates rewards for a family and penalties on childlessness as being of little avail because they leave untouched the real cause—namely, latter-day selfish avoidance of parenthood arising from love of pleasure. This phenomenon must be combated by inspiring universally higher ideals. Others have suggested modifying the conditions of employment of female school teachers so as to lessen the celibacy so common amongst them: seeking a similar dispensation for Roman Catholic priests has even been talked of. In Baden single women who have gone through a *bonâ-fide* betrothal (an engagement more binding than in England) with a combatant who by death or being on the "missing" list has been prevented from proceeding to marriage, are admitted to the style and title of a wife. Downright advocacy of polygamy is rare; more common is the recommendation to adhere to monogamy as one of the bases of society.

It will, of course, have been noticed that the tenor of most of the above openly discussed expedients antagonises more or less current sexual morality. That might have been foretold; for customary morality must always be modified in some degree by vital necessity. But a wide knowledge brings here, as everywhere, tolerance. But in Britain is (with an exception already mentioned) that wide knowledge anywhere to be found? The task of furthering due child-bearing in the next two decades is one in which, whoever else may participate, the sexuologist must.

